# Concurrent *parC* and *gyrA* fluoroquinolone resistance mutations and associated strains in *Mycoplasma genitalium* in Queensland, Australia

**DOI:** 10.1093/jac/dkad373

**Published:** 2023-12-15

**Authors:** Nicole G Ertl, Taylah K Anderson, Carolyn J Pardo, Toby I Maidment, Gerald L Murray, Catriona S Bradshaw, David M Whiley, Emma L Sweeney

**Affiliations:** The University of Queensland Centre for Clinical Research (UQCCR), Faculty of Medicine, The University of Queensland, Brisbane, Queensland, Australia; The University of Queensland Centre for Clinical Research (UQCCR), Faculty of Medicine, The University of Queensland, Brisbane, Queensland, Australia; The University of Queensland Centre for Clinical Research (UQCCR), Faculty of Medicine, The University of Queensland, Brisbane, Queensland, Australia; The University of Queensland Centre for Clinical Research (UQCCR), Faculty of Medicine, The University of Queensland, Brisbane, Queensland, Australia; The Department of Obstetrics and Gynaecology, University of Melbourne, Parkville, Victoria, Australia; Centre for Women’s Infectious Diseases, The Royal Women’s Hospital, Parkville, Victoria, Australia; Molecular Microbiology Research Group, Murdoch Children’s Research Institute, Parkville, Victoria, Australia; Melbourne Sexual Health Centre, Alfred Hospital and Central Clinical School, Monash University, Melbourne, Victoria, Australia; Central Clinical School, Monash University, Melbourne, Victoria, Australia; The University of Queensland Centre for Clinical Research (UQCCR), Faculty of Medicine, The University of Queensland, Brisbane, Queensland, Australia; Department of Microbiology, Pathology Queensland Central Laboratory, Brisbane, Queensland, Australia; The University of Queensland Centre for Clinical Research (UQCCR), Faculty of Medicine, The University of Queensland, Brisbane, Queensland, Australia

*Mycoplasma genitalium* is a cause of sexually transmitted infection that is associated with non-gonococcal urethritis and pelvic inflammatory disease.^1,2^ Over the past decade, *M. genitalium* has become increasingly resistant to recommended antimicrobials, including macrolides (>50% of *M. genitalium* globally) and fluoroquinolones (∼7.7%), with fluroquinolone resistance increasing along with associated treatment failures.^3,4^ Treatment failure with moxifloxacin is mediated by mutations in the fluoroquinolone resistance-determining region of the DNA topoisomerase (*parC*, amino acid positions S83 and D87) and DNA gyrase (*gyrA*, positions M95 and D99) genes.^4,5^ This has been further demonstrated by recent studies from Australia and Japan, showing an increased risk of moxifloxacin or sitafloxacin treatment failure where *M. genitalium* harboured both the ParC-S83I mutation (G248T DNA change) and a concurrent GyrA mutation affecting M95 (particularly M95I/G285A or G285T) or D99.^5,6^ Here, we explored the proportion of concurrent ParC and GyrA mutations in *M. genitalium* in Queensland, Australia, to better understand their co-occurrence and diagnostic value for resistance-guided treatment, and their potential links with specific *M. genitalium* genotypes.

*M. genitalium*-positive samples (*n* = 391; e.g. urine, urogenital and anal/rectal swabs) collected from male and female individuals between 2016 and 2021 in Queensland, Australia, were obtained from Pathology Queensland without corresponding clinical information about treatment success, and characterized for the presence of *parC* and *gyrA* mutations using established PCR assays and Sanger sequencing. A smaller representative subset (*n* = 139) was subjected to genotyping. Details are outlined in the Supplementary Methods (available as Supplementary data at *JAC* Online). Ethics approval was provided by the Children’s Health Queensland Human Research Ethics Committee (HREC/12/QRCH/139 and HREC/22/QCHQ/85249).

Samples from 326 patients (107 female, 214 male, 5 not specified) were included. Determination of the proportion of samples with *parC* and *gyrA* mutations was based on a single sample per patient (*n* = 326 samples), except one patient exhibiting reinfection >2 years later (*n* = 327/391 samples). A further 4% of samples were excluded from further analysis after repeat sequencing failure of one or both loci (Table S1). Analysis of the remaining 96% (314/327) of samples characterized for both *parC* and *gyrA* genes showed that the M95I (G285A or G285T) mutation was the most common GyrA mutation observed, found in 29.6% (93/314) of samples, while 2.2% (7/314) of samples carried a D99 mutation. Interestingly, 2.9% (9/314) of samples contained ‘mixed’ susceptibility populations, with five harbouring both GyrA WT and a mutation (e.g. M95I, A96T, F108I) within the same sample, and four samples harbouring single/dual GyrA mutations at two nucleotide positions (Figure 1a, Table S2).

**Figure 1.**
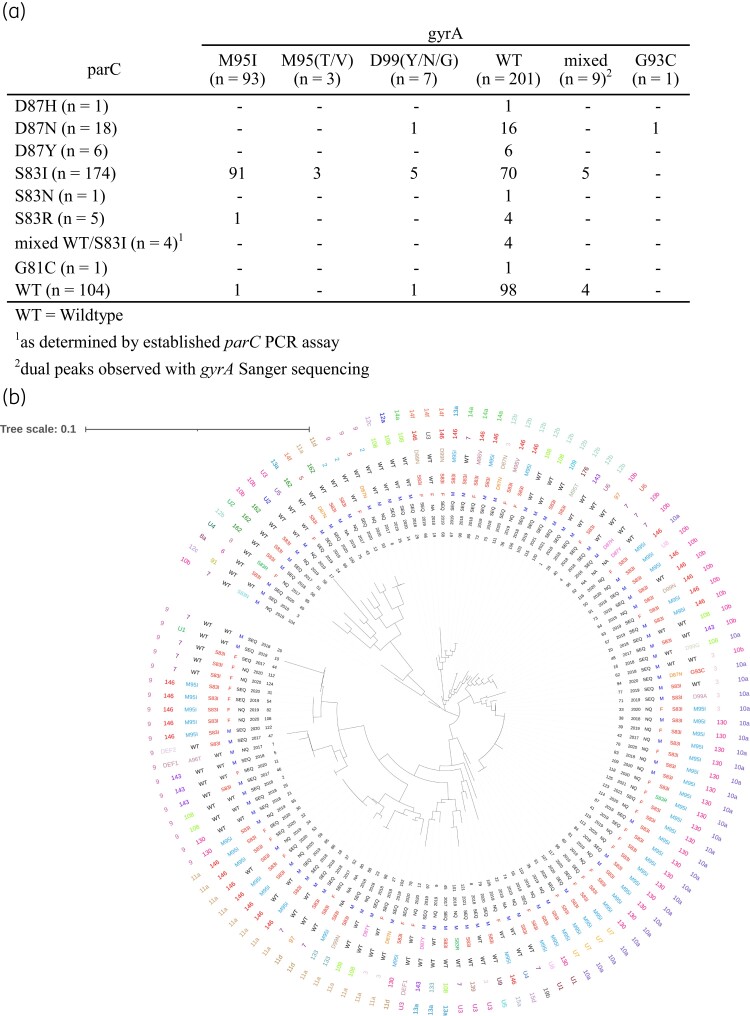
Concurrent ParC and GyrA mutations as determined by PCR and Sanger sequencing (a) and their respective MG191 and MG309 STs depicted in a neighbour-joining phylogenetic tree (b) in *M. genitalium* samples from Queensland, Australia. Data (from innermost to outermost) depicted correspond to sample ID, collection year and location (SEQ = South East Queensland; NQ = Northern Queensland; NA = unknown), gender (NA = unknown), ParC and GyrA mutations, and MG191 and MG309 loci. This figure appears in colour in the online version of *JAC* and in black and white in the print version of *JAC*.

Of the 55.4% (174/314) samples with a ParC-S83I (G248T) mutation, 56.9% (99/174) also had a single concurrent mutation in GyrA, with M95I being the most common (52.3%; 91/174; G285A = 90, G285T = 1). GyrA mutations were rare in ParC WT samples (5.8%; 6/104), or samples harbouring non-S83I mutations in ParC (9.4%; 3/32) (Figure 1a, Table S1, Supplementary Results).

Genotyping (MG191 and MG309 loci) was available for 135/139 samples with available *parC* and *gyrA* sequences. Of 125 individual patient samples, we identified 28 MG191 and 21 MG309 STs, including 9 novel MG191 and 6 novel MG309 STs (Figure 1b, Table S1, Table S2). The most common MG191 STs were 130 and 146 (22/125; 17.6% and 26/125; 20.8%, respectively), which frequently harboured dual ParC-S83I/GyrA-M95I mutations (Figure 1b).

Genotypes were assigned based on combined MG191 and MG309 data, with 71 genotypes from 125 individual patient samples (1–18 samples per genotype) (Figure 1b). Of these, 16 harboured dual ParC-S83I/GyrA-M95I mutations, with genotype 35 (*n* = 18 samples) being the most common, which exclusively harboured dual ParC and GyrA mutations. Of the remaining 15 genotypes harbouring concurrent ParC-S83I and GyrA-M95I mutations, almost all of these belonged to MG191 ST130 or ST146 and had differing MG309 STs (see Table S1 and Supplementary Results).

In summary, consistent with recent findings in Australia and Japan,^5–8^ GyrA-M95I was the most common GyrA mutation observed, with the majority of GyrA mutations co-occurring with ParC-S83I. The GyrA-M95I mutation was rarely found in samples considered to be ParC WT or non-S83I (2.2%; 2/93). Combined with results from Hamasuna *et al*.,^9^ which showed elevated moxifloxacin MICs (≥2 mg/L) in *M. genitalium* strains with concomitant ParC-S83I and GyrA mutations, and recent studies linking the presence of concurrent ParC-S83I and GyrA mutations to significantly lower cure rates with sitafloxacin and moxifloxacin,^5,6^ this study further highlights the utility of diagnostic tests that include both ParC-S83I and GyrA-M95I for precision treatment of *M. genitalium*.

Interestingly, while previous studies suggested no association between genotype and antimicrobial resistance in *M. genitalium*,^8,10^ the distribution of concurrent ParC and GyrA mutations among *M. genitalium* genotypes in this study suggests enrichment of the dual ParC and GyrA mutations in the MG191 STs 130 and 146. These key STs were found among male and female patients within the study, and across geographically distinct locations, suggesting these highly resistant strains may be circulating among large/complex sexual networks.

## Supplementary Material

dkad373_Supplementary_Data

## Transparency declarations

Unrelated to this study, C.S.B., D.M.W., E.L.S. and G.L.M. report receiving funding support and diagnostic kits from SpeeDx Pty Ltd for research on *M. genitalium*. SpeeDx played no role in the conception, planning, analysis or decision to publish this work. G.L.M. also reports receiving TIB MolBiol diagnostic kits for his laboratory unrelated to this study. C.S.B. has advised a number of industries over the years including Nabriva, GSK and Roche Pty Ltd, but has not received payment for this advisory role, and reports a leadership/fiduciary role in the ISSTDR Board. N.G.E., C.J.P., T.I.M. and T.K.A. report no conflicts of interest. All authors have submitted the ICMJE Form for Disclosure of Potential Conflicts of Interest.

## Supplementary data

Supplementary Methods, Results, Figures S1–S2 and Tables S1–S2 are available as Supplementary data at *JAC* Online.
