# Supplementary material for: Concurrent parC and gyrA fluoroquinolone resistance mutations and associated strains in Mycoplasma genitalium in Queensland, Australia
Source: J Antimicrob Chemother. 2023 Dec 15;79(2):467–9. doi: 10.1093/jac/dkad373 (PMC10832590; doi:10.1093/jac/dkad373)
Supplement: dkad373_Supplementary_Data [file dkad373_supplementary_data.zip › Supplementary file.docx]

**Supplementary file**

# METHODS

*Mycoplasma genitalium*-positive samples (n=391) were collected in 2016 (n=1), 2017 (n=43), 2018 (n=72), 2019 (n=142), 2020 (n=110) and 2021 (n=19), as well as n=4 with unknown collection date (Table S1). These samples were received by Pathology Queensland from health clinics without clinical information and will therefore contain samples from a diverse range of patients (both pre-treated, common among STI clinic populations, and treatment naïve). Swab samples were received in Cobas buffer, DNA extracted from all samples using Roche Cobas or MagNA Pure 96 extraction platforms depending on the originating pathology laboratory, and extracts stored at -20°C until use. Samples were first tested with a previously published MgPa assay ^1^ to confirm *M. genitalium* load for subsequent analyses. Samples were then characterised for the presence of *parC* mutations using Sanger sequencing ^2^ or probe-based PCR assays,^3, 4^ followed by *gyrA* Sanger sequencing.^2^ In addition, a smaller representative subset of samples (n = 139) were subjected to genotyping, targeting the MG191 and MG309 gene loci.^5^ With previous studies showing no substantial link between antimicrobial resistance and genotype,^6, 7^ this genotyping was carried out to assess the heterogeneity of genotypes and to determine if the same trend could be observed for the dual mutants of this data set. For this, a representative genotyping subset was chosen from the larger *M. genitalium* (n=391) dataset with the aim to capture samples harbouring the different ParC with GyrA combinations and their occurrence, as seen in the broader data set. The subset for genotyping included ParC-D87(H/N/Y) with GyrA wildtype or mutation (D99Y or G93C) (n=10), ParC mutation not characterised with GyrA wildtype (n=1), ParC-S83I with GyrA-M95I (n=50), ParC-S83I with non-M95I GyrA (mixed/D99N/M95T/M95V) (n=13), ParC-S83I with GyrA wildtype (n=24), non-S83I (S83N/R) ParC with GyrA-M95I or wildtype (n=4), and ParC wildtype with either GyrA wildtype (n=32) or GyrA mutation (mixed/D99G/M95I) (n=5). All PCR products for sequencing were submitted to the Australian Genome Research Facility (AGRF; Brisbane, Australia), and the resulting nucleotide sequences compared to *M. genitalium* strain G37 (GenBank accession no. NC_000908.2) for *parC* and *gyrA* SNP detection. MG191 sequence type (based on specific single nucleotide polymorphisms) and MG309 sequence type (based on specific short tandem repeats) assignment was based on previously published sequence types,^5, 6, 8, 9^ and genotypes assigned to each sample based on concatenated MG191 and MG309 sequences. Neighbour joining phylogenetic trees were prepared from ClustalW alignments using the Geneious Prime^®^ bioinformatics software (version 2022.0.2) and visualised using iTol.^10^ Individual MG191 and MG309 loci were then concatenated to show the relationship among strains genotyped in Queensland.

# RESULTS

A total of 391 samples, originating from 326 patients were analysed in this study, with the proportion of samples with *parC* and *gyrA* mutations based on a single sample per patient, except for one patient with an apparent re-infection >2 years later (n=327/391 samples). Of those, there was a relative even distribution of samples from South East Queensland (n=192) and Northern Queensland (n=117), with 18 samples from unspecified regions (Table S1). In contrast, most of the 327 samples came from patients 21-25 years old (n=89), followed by patients aged 26-30 (n=79), 31-35 (n=50), 36-40 (n=34) and 18-20 (n=26) with all other age groups contributing <20 samples each.

**ParC and GyrA:**

PCR and Sanger sequencing of the 327 samples resulted in characterisation of 314 samples for both *parC* and *gyrA* genes, with 4% (13) of samples excluded from further analysis after repeated sequencing failure of one or both loci (Table S1). Interestingly, analysis of the *gyrA* Sanger sequencing chromatographs showed double peaks in 2.9% (9/314) of samples at nucleotide positions 272, 285, 286, 296, 322 and 356 (conferring changes at amino acid positions 91, 95, 96, 99, 108 and 119, respectively), indicating “mixed” susceptibility populations (Tables 1, S2).

While concurrent GyrA mutations were rare in samples that did not harbour ParC-S83I, GyrA-M95I was also found in 20% (1/5) of ParC-S83R (A247C) samples, and 11.1% (2/18) of samples with a ParC-D87N (G259A) change also harboured non-M95I GyrA mutations.

**Genotyping:**

Individual phylogenetic trees for the MG191 and MG309 loci are presented in Figures S1 and S2 and show ST146 and ST130, as well as ST10a and ST9 to be the most common MG191 and MG309 STs, respectively. Genotypes were then assigned to each sample based on combined MG191 and MG309 data, except in the instances where samples were uncharacterised for all four loci (ParC, GyrA, MG191 and/or MG309), or samples with multiple results for MG191 or MG309.

**Genotyping and antimicrobial resistance among patients sampled over time:**

Of the 326 patients, 43 patients had multiple samples (between 2 to 5 samples) taken over time that could be characterised for both, ParC and GyrA. Assessment of those consecutive samples from the same patient showed that there was no change in ParC and GyrA mutations in 36/43 patients over time, with consistency in fluoroquinolone resistance mutations for up to ~10 months (Table S1). Of those, and where consecutive genotyping data was available, we also identified the same *M. genitalium* genotype in 4/36 patient samples, suggestive that these infections were likely chronic in nature, and less likely to be as a consequence of reinfection. In contrast, one patient had results consistent with reinfection with a different *M. genitalium* strain; with differences in MG191 (ST146 to 130) and MG309 (ST10a to U6) genotyping data, as well as concurrent ParC and GyrA mutations observed. Interestingly, the first sample for this patient (sample 116.1) indicated the presence of a GyrA dual peak in sequencing chromatographs (single with dual GyrA mutant) with a concurrent ParC-S83I mutation, while the sample collected ~3 months later showed a dual ParC wildtype/GyrA-M95I (Table S1).

# Supplementary figures


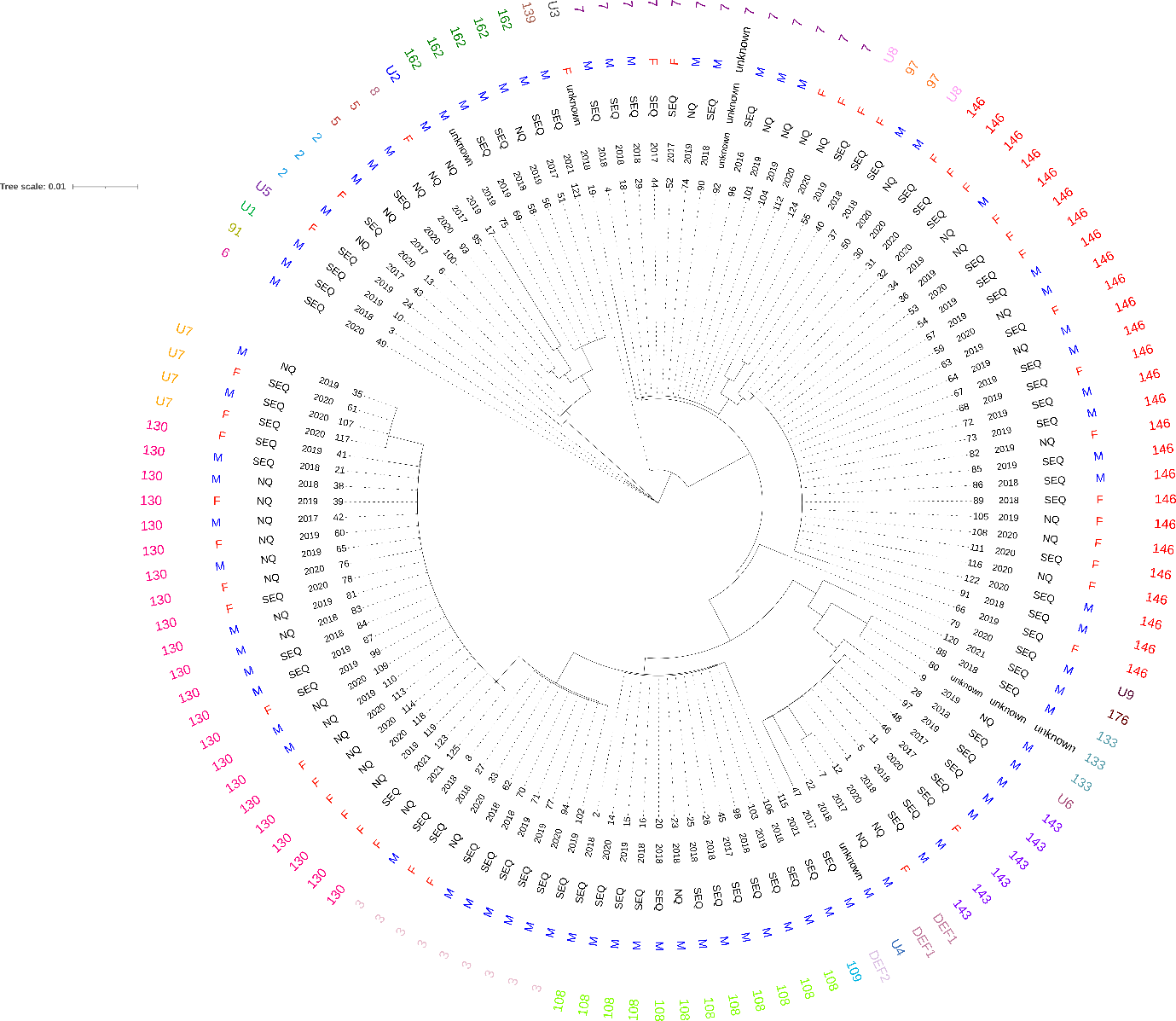


**Figure S1** Neighbour joining phylogenetic tree of MG191 sequence types. Tree was prepared in Geneious Prime^®^ bioinformatics software from ClustalW aligned sequences and include demographic data where available and visualised using iTOL. Data shown in the tree (from innermost to outermost) include sample ID, collection year and location (SEQ = South East Queensland; NQ = Northern Queensland), gender and MG191 sequence type.


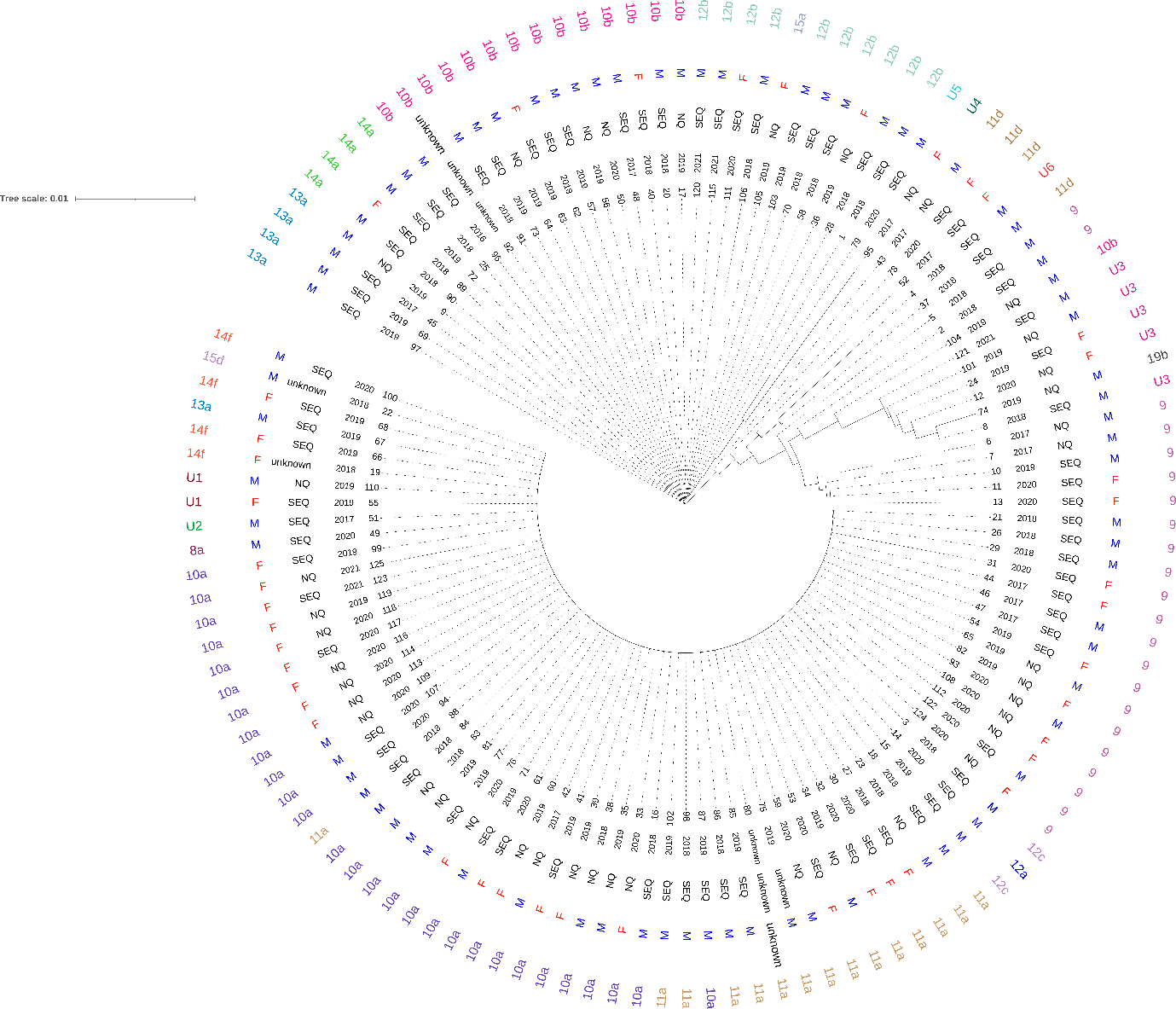


**Figure S2** Neighbour joining phylogenetic tree of MG309 sequence types. Tree was prepared in Geneious Prime^®^ bioinformatics software from ClustalW aligned sequences and include demographic data where available and visualised using iTOL. Data shown in the tree (from innermost to outermost) include sample ID, collection year and location (SEQ = South East Queensland; NQ = Northern Queensland), gender and MG309 sequence type.

# References

**1** Jensen JS, Björnelius E, Dohn B et al. Use of TaqMan 5' nuclease real-time PCR for quantitative detection of *Mycoplasma genitalium* DNA in males with and without urethritis who were attendees at a sexually transmitted disease clinic. *J Clin Microbiol* 2004; **42**: 683-92.

**2** Deguchi T, Maeda S-I, Tamaki M et al. Analysis of the *gyrA* and *parC* genes of *Mycoplasma genitalium* detected in first-pass urine of men with non-gonococcal urethritis before and after fluoroquinolone treatment. *J Antimicrob Chemother* 2001; **48**: 735-44.

**3** Tickner JA, Bradshaw CS, Murray GL et al. Novel probe-based melting curve assays for the characterization of fluoroquinolone resistance in *Mycoplasma genitalium*. *J Antimicrob Chemother* 2022; **77**: 1592-9.

**4** Sweeney EL, Lowry K, Bletchly C et al. *Mycoplasma genitalium* infections can comprise a mixture of both fluoroquinolone-susceptible and fluoroquinolone-resistant strains. *J Antimicrob Chemother* 2021; **76**: 887-92.

**5** Ma L, Taylor S, Jensen JS et al. Short tandem repeat sequences in the *Mycoplasma genitalium* genome and their use in a multilocus genotyping system. *BMC Microbiol* 2008; **8**: 130.

**6** Sweeney EL, Tickner J, Bletchly C et al. Genotyping of *Mycoplasma genitalium* suggests *de novo* acquisition of antimicrobial resistance in Queensland, Australia. *J Clin Microbiol* 2020; **58**: e00641-20.

**7** Chua T-P, Bodiyabadu K, Machalek DA et al. Prevalence of *Mycoplasma genitalium* fluoroquinolone-resistance markers, and dual-class-resistance markers, in asymptomatic men who have sex with men. *J Med Microbiol* 2021; **70**: 001429.

**8** Pond MJ, Nori AV, Witney AA et al. High prevalence of antibiotic-resistant *Mycoplasma genitalium* in nongonococcal urethritis: the need for routine testing and the inadequacy of current treatment options. *Clin Infect Dis* 2014; **58**: 631-7.

**9** Piñeiro L, Idigoras P, Cilla G. Molecular typing of *Mycoplasma genitalium*-positive specimens discriminates between persistent and recurrent infections in cases of treatment failure and supports contact tracing. *Microorganisms* 2019; **7**: 609.

**10** Letunic I, Bork P. Interactive Tree Of Life (iTOL) v5: an online tool for phylogenetic tree display and annotation. *Nucleic Acids Res* 2021; **49**: W293-W6.
