# Supplementary material for: Concurrent parC and gyrA fluoroquinolone resistance mutations and associated strains in Mycoplasma genitalium in Queensland, Australia
Source: J Antimicrob Chemother. 2023 Dec 15;79(2):467–9. doi: 10.1093/jac/dkad373 (PMC10832590; doi:10.1093/jac/dkad373)
Supplement: dkad373_Supplementary_Data [file dkad373_supplementary_data.zip › Table S1.pdf]

Table S1 *Mycoplasma genitalium* positive samples from Queensland and their fluoroquionolone resistance markers and genotypes as determined by PCR and Sanger sequencing. Combined genotype numbers were based on the MG191 and MG309 loci for *M. genitalium.*

Sample ID	Collection location	Gender	Age group	Specimen	Date of collection	AA ParC change*	DNA gyrA change*	AA GyrA change*	Genotyping MG191	Genotyping MG309	Combined genotype number
1	SEQ	M	26-30	Urine	Apr-2018	Wildtype	WT	Wildtype	143	12b	45
2	SEQ	M	36-40	Rectal swab	Apr-2018	Wildtype	WT	Wildtype	108	9	24
3	SEQ	M	21-25	Urine	Mar-2018	Wildtype	WT	Wildtype	91	12c	20
4	SEQ	M	31-35	Urine	Feb-2018	Wildtype	WT	Wildtype	7	U6	18
5	SEQ	M	26-30	Rectal swab	Feb-2018	Wildtype	WT	Wildtype	143	9	43
6	NQ	M	21-25	Urine	May-2017	Wildtype	WT	Wildtype	2	9	1
7	NQ	M	31-35	Urine	Jul-2017	Wildtype	G286A / WT	A96T / Wildtype	DEF1	9	63
7.1	NQ	M	31-35	Urine	Apr-2017	Wildtype	WT / G286A	Wildtype / A96T	-	-	-
8	SEQ	M	26-30	Urethral swab	Feb-2018	Wildtype	WT	Wildtype	3	U3	8
9	NQ	M	26-30	Rectal swab	Jan-2019	Wildtype	WT	Wildtype	133	13a	41
10	SEQ	M	41-45	Urine	Sep-2019	Wildtype	WT	Wildtype	U1	9	66
11	SEQ	F	18-20	Lower vaginal swab	Nov-2020	Wildtype	WT	Wildtype	143	9	43
12	NQ	F	18-20	Urine	Oct-2020	Wildtype	WT	Wildtype	DEF1	U3	64
13	SEQ	F	26-30	High vaginal swab	Dec-2020	Wildtype	WT	Wildtype	2	9	1
14	SEQ	M	46-50	Urethral swab	Nov-2020	Wildtype	WT	Wildtype	108	12a	28
15	SEQ	M	21-25	Urine	Feb-2019	Wildtype	WT	Wildtype	108	12c	30
16	SEQ	M	46-50	Urine	Feb-2018	Wildtype	A296G	D99G	108	10a	25
17	NQ	M	26-30	unknown	Jan-2019	Wildtype	WT	Wildtype	U2	10b	67
18	SEQ	M	21-25	Urine	Dec-2018	Wildtype	WT	Wildtype	7	11a	14
19	Other region not specified	F	36-40	unknown	Dec-2018	Wildtype	WT	Wildtype	U3	14f	68
20	SEQ	M	21-25	Rectal swab	Nov-2018	Wildtype	WT	Wildtype	108	10b	26
21	SEQ	M	21-25	Urine	Aug-2018	Wildtype	WT	Wildtype	130	9	34
22	Other region not specified	M	26-30	Urine	Oct-2018	Wildtype	WT	Wildtype	U4	15d	69
23	NQ	M	36-40	Urine	Oct-2018	Wildtype	WT	Wildtype	108	11a	27
24	SEQ	F	21-25	Vaginal swab	Feb-2019	Wildtype	WT	Wildtype	U5	U3	70
24.1	SEQ	F	21-25	Vaginal swab	Feb-2019	Wildtype	WT	Wildtype	2	U3	3
25	SEQ	M	21-25	Rectal swab	Jan-2018	Wildtype	WT	Wildtype	108	14a	32
26	SEQ	M	>55	Urine	Jun-2018	Wildtype	WT	Wildtype	108	9	24
27	SEQ	F	21-25	Urine	May-2018	Wildtype	WT	Wildtype	3	11a	6
27.1	SEQ	F	21-25	unknown	May-2018	Wildtype	WT	Wildtype	-	-	-
28	SEQ	M	26-30	Urine	Apr-2018	Wildtype	WT	Wildtype	U6	12b	71
29	SEQ	M	21-25	Urine	Jan-2018	Wildtype	WT	Wildtype	7	9	12
30	SEQ	F	36-40	Cervical swab	Nov-2020	S83I	G285A	M95I	146	11a	50
31	SEQ	F	36-40	Vaginal swab	Oct-2020	S83I	G285A / G285A & G286A	M95I / M95I and A96T	146	9	47
31.1	SEQ	F	31-35	Urine	Jul-2020	S83I	G285A	M95I	-	-	-
32	SEQ	F	36-40	Cervical swab	Oct-2020	S83I	G285A	M95I	146	11a	50
33	NQ	F	21-25	Vaginal swab	Aug-2020	S83I	G285A	M95I	3	10a	4
34	NQ	M	21-25	Urine	Mar-2019	S83I	WT	Wildtype	146	11a	50
35	NQ	M	31-35	Urine	Feb-2019	S83I	G285A	M95I	U7	10a	72
36	NQ	F	26-30	Vaginal swab	Mar-2019	S83I	G285A	M95I	146	12b	51
37	SEQ	M	26-30	Urine	Dec-2018	S83I	WT	Wildtype	97	11d	22
37.1	SEQ	M	26-30	Urine	Oct-2018	S83I	WT	Wildtype	97	11d/12b	not characterised
37.2	SEQ	M	26-30	Urine	Jan-2019	S83I	WT	Wildtype	-	-	-
37.3	SEQ	M	26-30	Urine	Nov-2018	S83I	WT	Wildtype	-	-	-
38	NQ	M	26-30	Urine	Dec-2018	S83I	G285A	M95I	130	10a	35
39	NQ	F	21-25	High vaginal swab	Mar-2019	S83I	G285A	M95I	130	10a	35
39.1	NQ	F	21-25	No site specified	Oct-2018	S83I	G285A	M95I	-	-	-
40	SEQ	F	21-25	Urine	Dec-2018	S83I	WT	Wildtype	97	10b	21
41	SEQ	F	18-20	Cervical swab	Feb-2019	S83I	G285A	M95I	130	10a	35
42	NQ	M	26-30	Urine	Jun-2017	S83I	G285A	M95I	130	10a	35
43	NQ	M	26-30	Urine	Oct-2017	S83I	WT	Wildtype	2	11d	2
44	SEQ	F	21-25	Cervical swab	Sep-2017	S83I	WT	Wildtype	7	9	12
45	SEQ	M	26-30	Urine	Sep-2017	S83I	WT	Wildtype	108	13a	31
46	SEQ	M	31-35	Urine	Sep-2017	S83I	WT	Wildtype	143	9	43
47	SEQ	M	26-30	Urine	Jun-2017	S83I	WT	Wildtype	DEF2	9	65
48	SEQ	M	26-30	Rectal swab	Sep-2017	S83I	WT	Wildtype	143	10b	44
49	SEQ	M	31-35	Urine	Nov-2020	S83I	WT	Wildtype	6	8a	11
50	NQ	M	31-35	Urine	Nov-2020	S83I	G285A	M95I	U8	10b	73
50.1	NQ	M	31-35	Urine	Jan-2020	S83I	G285A	M95I	-	-	-
50.2	NQ	M	31-35	Urine	Apr-2020	S83I	G285A	M95I	-	-	-
51	SEQ	M	31-35	Urine	Sep-2017	S83I	WT	Wildtype	162	U2	61
51.1	SEQ	M	31-35	unknown	Jan-2018	S83I	WT	Wildtype	-	-	-
52	SEQ	F	41-45	Vaginal swab	May-2017	S83I	WT / T322A	Wildtype / F108I	7	11d	15
53	SEQ	F	31-35	Urine	Jun-2020	S83I	G285A	M95I	146	11a	50
54	SEQ	F	21-25	Vaginal swab	Sep-2019	S83I	G285A	M95I	146	9	47
54.1	SEQ	F	21-25	Cervical swab	May-2019	S83I	G285A	M95I	-	-	-
54.2	SEQ	F	21-25	Cervical swab	Apr-2019	S83I	G285A	M95I	-	-	-

54.3	SEQ	F	21-25	Cervical swab	Jul-2019	S83I	G285A	M95I	-	-	-
54.4	SEQ	F	21-25	Vaginal swab	Jul-2019	S83I	G285A	M95I	-	-	-
55	SEQ	F	21-25	Vaginal swab	Aug-2019	S83I	G285A	M95I	U8	U1	74
55.1	SEQ	F	21-25	unknown	Jul-2019	S83I	G285A	M95I	-	-	-
55.2	SEQ	F	21-25	Urine	Aug-2019	S83I	G285A	M95I	-	-	-
56	NQ	M	21-25	Urine	Aug-2019	S83I	WT	Wildtype	162	10b	57
57	SEQ	M	31-35	unknown	Nov-2019	S83I	G285A	M95I	146	10b	49
58	SEQ	M	41-45	Urine	Nov-2018	Wildtype	WT	Wildtype	162	12b	59
59	NQ	M	21-25	Urine	Jun-2020	S83I	G285A	M95I	146	11a	50
59.1	NQ	M	21-25	Urine	Jun-2020	S83I	G285A	M95I	-	-	-
59.2	NQ	M	21-25	Urine	Aug-2020	S83I	G285A	M95I	-	-	-
60	NQ	F	21-25	Cervical swab	Oct-2019	S83I	G285A	M95I	130	10a	35
61	SEQ	F	31-35	Vaginal swab	Jun-2020	S83I	G285A	M95I	U7	10a	72
61.1	SEQ	F	31-35	Vaginal swab	Sep-2020	S83I	G285A	M95I	-	-	-
61.2	SEQ	F	31-35	Urine	Sep-2020	S83I	G285A	M95I	-	-	-
61.3	SEQ	F	31-35	Vaginal swab	Aug-2020	S83I	G285A	M95I	-	-	-
61.4	SEQ	F	31-35	unknown	Aug-2020	S83I	Undetermined	not characterised	-	-	-
62	SEQ	M	21-25	Urine	Nov-2018	Wildtype	WT	Wildtype	3	10b	5
63	SEQ	F	21-25	Urine	Oct-2019	S83I	G285A	M95I	146	10b	49
63.1	SEQ	F	21-25	Vaginal swab	Dec-2019	S83I	G285A	M95I	Undetermined	10b	not characterised
63.2	SEQ	F	21-25	Urine	Dec-2019	S83I	G285A	M95I	146	10b	49
63.3	SEQ	F	21-25	Vaginal swab	Aug-2020	S83I	G285A	M95I	-	-	-
64	NQ	M	51-55	Urine	May-2019	S83I	G295A	D99N	146	10b	49
65	NQ	M	31-35	Urine	Aug-2019	S83I	G285A	M95I	130	9	34
65.1	NQ	M	31-35	Urine	Nov-2019	S83I	G285A	M95I	130	10a	35
66	SEQ	F	26-30	Vaginal swab	Jun-2019	S83I	G295A	D99N	146	14f	55
67	SEQ	M	41-45	Urine	May-2019	S83I	G285T	M95I	146	13a	53
68	SEQ	F	26-30	Vaginal swab	May-2019	S83I	G295A	D99N	146	14f	55
69	SEQ	M	36-40	Urine	Jun-2019	S83I	WT	Wildtype	162	13a	60
69.1	SEQ	M	36-40	Urine	May-2019	S83I	WT	Wildtype	-	-	-
69.2	SEQ	M	36-40	Urine	Aug-2019	S83I	WT	Wildtype	-	-	-
70	SEQ	M	26-30	Urine	Nov-2018	D87N	G295T	D99Y	3	12b	7
71	SEQ	M	51-55	Urine	Aug-2019	S83I	A296C / A296C and T322A	D99A / D99A and F108I	3	10a	4
72	SEQ	M	26-30	Urine	Aug-2019	S83I	G285A	M95I	146	14a	54
73	SEQ	M	41-45	Urine	Jun-2019	S83I	WT	Wildtype	146	10b	49
74	NQ	M	21-25	Urine	Mar-2019	S83I	WT	Wildtype	7	19b	16
75	Other region not specified	M	31-35	Urine	Nov-2019	S83I	WT	Wildtype	162	11a	58
76	NQ	F	18-20	Vaginal swab	Jun-2020	S83I	G285A	M95I	130	10a	35
77	SEQ	M	51-55	Urine	Jan-2019	S83I	WT	Wildtype	3	10a	4
77.1	SEQ	M	51-55	Penile swab	Jan-2019	S83I	WT	Wildtype	-	-	-
78	SEQ	F	26-30	Cervical swab	Nov-2020	S83I	G285A	M95I	130	11d	37
78.1	SEQ	F	21-25	Cervical swab	Oct-2018	Wildtype	WT	Wildtype	-	-	-
79	SEQ	M	18-20	Urine	Oct-2020	S83I	WT	Wildtype	U9	U5	75
80	unknown	unknown	unknown	unknown	unknown	S83I	G295A	D99N	133	11a	40
81	NQ	M	21-25	Urine	Feb-2019	S83I	G285A	M95I	130	10a	35
82	NQ	F	26-30	Vaginal swab	Aug-2019	S83I	G285A	M95I	146	9	47
83	NQ	M	21-25	Urine	Dec-2018	S83I	G285A	M95I	130	10a	35
83.1	NQ	M	21-25	Urine	Jan-2019	S83I	G285A	M95I	-	-	-
84	SEQ	M	26-30	Penile swab	Sep-2018	S83I	G285A	M95I	130	10a	35
84.1	SEQ	M	26-30	Urine	Sep-2018	S83I	G285A	M95I	130	10a	35
84.2	SEQ	M	26-30	Urine	Oct-2018	S83I	G285A	M95I	130	10a	35
85	SEQ	M	36-40	Urine	Feb-2019	Wildtype	WT / G285A	Wildtype / M95I	146	11a	50
85.1	SEQ	M	36-40	Urine	Apr-2019	S83I	G285A	M95I	-	-	-
86	SEQ	M	26-30	Urine	Jun-2018	S83I	G285A	M95I	146	11a	50
86.1	SEQ	M	26-30	Urine	Jul-2018	S83I	G285A	M95I	146	11a	50
87	SEQ	M	31-35	Urethral swab	Jan-2019	S83I	G285A	M95I	130	10a	35
88	SEQ	M	31-35	Urine	Jun-2018	S83I	G295A	D99N	133	11a	40
89	SEQ	F	18-20	High vaginal swab	Apr-2018	S83I	A283G	M95V	146	14a	54
90	SEQ	M	21-25	Rectal swab	Feb-2018	S83I	WT	Wildtype	7	14a	15
91	SEQ	M	18-20	Urine	Feb-2018	S83I	G285A	M95I	146	10b	49
92	unknown	unknown	unknown	unknown	unknown	D87Y	WT	Wildtype	7	10b	13
93	NQ	M	26-30	Urine	Aug-2020	D87N	WT	Wildtype	5	9	9
94	SEQ	M	41-45	Urine	Aug-2020	D87N	G277T	G93C	3	10a	4
94.1	SEQ	M	41-45	Urine	May-2019	D87N	G277T	G93C	3	Undetermined	not characterised
95	NQ	F	21-25	Urine	Feb-2017	S83R	WT	Wildtype	8	U4	19
96	SEQ	M	26-30	Urine	Dec-2016	D87H	WT	Wildtype	7	10b	13
97	SEQ	M	46-50	Urethral swab	Jan-2019	D87Y	WT	Wildtype	143	13a	46
98	SEQ	M	>55	Urine	Oct-2018	D87Y	WT	Wildtype	108	11a	27
99	SEQ	F	26-30	Cervical swab	Sep-2019	S83I	G285A	M95I	130	10a	35

100	SEQ	M	21-25	Urine	Jan-2020	D87N	WT	Wildtype	5	14f	10
101	NQ	M	41-45	Anal swab	Jul-2019	S83R	WT	Wildtype	7	U3	17
102	SEQ	M	31-35	Rectal swab	Jan-2019	D87N	WT	Wildtype	3	11a	6
103	SEQ	M	21-25	Urine	Mar-2019	Wildtype	WT	Wildtype	108	12b	29
104	NQ	M	36-40	Urine	Aug-2019	S83N	WT	Wildtype	7	10b	13
104.1	NQ	M	36-40	Urine	Jul-2019	S83R	WT	Wildtype	-	-	-
105	NQ	F	31-35	unknown	Mar-2019	Wildtype	G285A	M95I	146	15a	56
106	SEQ	M	>55	Urethral swab	Aug-2018	Wildtype	WT	Wildtype	108	12b	29
107	SEQ	M	31-35	Urethral swab	Apr-2020	S83I	G285A	M95I	U7	10a	72
108	NQ	F	31-35	Cervical swab	Apr-2020	S83I	G285A	M95I	146	9	47
109	NQ	M	26-30	Urine	Apr-2020	S83I	G285A	M95I	130	10a	35
109.1	NQ	M	26-30	Urine	May-2020	S83I	G285A	M95I	130	10a	35
109.2	NQ	M	26-30	Urethral swab	May-2020	S83I	G285A	M95I	-	-	-
110	NQ	M	26-30	Urine	Aug-2019	S83I	G285A	M95I	130	U1	38
110.1	NQ	M	26-30	Urine	Sep-2019	S83I	G285A	M95I	130	10b	36
111	SEQ	F	31-35	Lower vaginal swab	Feb-2020	S83I	A283G	M95V	146	12b	52
112	NQ	F	18-20	High vaginal swab	Feb-2020	S83I	WT	Wildtype	7	9	12
113	NQ	F	26-30	unknown	Feb-2020	S83I	G285A / G285A & T322A	M95I / M95I and F108I	130	10a	35
114	NQ	F	21-25	Vaginal swab	Aug-2020	S83I	G285A	M95I	130	10a	35
115	SEQ	M	26-30	Urine	May-2021	S83I	WT	Wildtype	109	12b	33
116	NQ	F	18-20	Cervical swab	Jul-2020	S83I	G285A / G285A & T322A	M95I / M95I and F108I	146	10a	48
116.1	NQ	F	18-20	Cervical swab	Nov-2020	Wildtype	G285A	M95I	130	U6	39
116.2	NQ	F	18-20	Cervical swab	Sep-2020	S83I	G285A	M95I	-	-	-
117	SEQ	F	21-25	Vaginal swab	Aug-2020	S83I	G285A	M95I	U7	10a	72
118	NQ	F	31-35	Vaginal swab	Jul-2020	S83I	G285A	M95I	130	10a	35
119	NQ	F	21-25	High vaginal swab	Aug-2019	S83I	G285A	M95I	130	10a	35
120	SEQ	M	36-40	Urine	Jan-2021	S83I	T284C	M95T	176	12b	62
121	SEQ	M	26-30	Urine	Mar-2021	S83I	WT	Wildtype	139	U3	42
122	SEQ	M	26-30	Urine	Nov-2020	S83I	G285A	M95I	146	9	47
123	SEQ	F	31-35	unknown	Jan-2021	S83I	G285A	M95I	130	10a	35
123.1	SEQ	F	31-35	Cervical swab	Nov-2020	S83I	G285A	M95I	-	-	-
124	NQ	F	31-35	Vaginal swab	Oct-2020	S83I	WT	Wildtype	7	9	12
125	NQ	F	21-25	Cervical swab	Feb-2021	S83R	G285A	M95I	130	10a	35
126	Other region not specified	M	26-30	Urine	Mar-2017	Wildtype	WT	Wildtype	-	-	-
127	Other region not specified	F	41-45	Urine	Nov-2019	Wildtype	WT	Wildtype	-	-	-
128	Other region not specified	M	18-20	Urine	Jan-2019	Wildtype	WT	Wildtype	-	-	-
129	Other region not specified	M	26-30	Urine	Dec-2018	S83I	WT	Wildtype	-	-	-
130	Other region not specified	M	36-40	Urine	Jun-2018	Wildtype	WT	Wildtype	-	-	-
131	Other region not specified	M	36-40	Urine	Sep-2019	S83I	WT	Wildtype	-	-	-
132	NQ	M	21-25	Urine	Oct-2020	Wildtype	WT	Wildtype	-	-	-
133	NQ	M	26-30	Urine	Jul-2019	S83I	G285A	M95I	-	-	-
134	NQ	M	26-30	Urine	Aug-2017	S83I	WT	Wildtype	-	-	-
135	NQ	F	21-25	Urine	Jul-2017	Wildtype	WT	Wildtype	-	-	-
136	NQ	M	36-40	Urine	Feb-2017	D87N	WT	Wildtype	-	-	-
137	NQ	F	21-25	Lower vaginal swab	Oct-2020	S83I	G285A	M95I	-	-	-
138	NQ	F	36-40	Urine	Mar-2017	Wildtype	WT	Wildtype	-	-	-
139	SEQ	F	31-35	Urine	Aug-2018	Wildtype	WT	Wildtype	-	-	-
140	SEQ	F	26-30	Lower vaginal swab	Oct-2020	S83I	G285A	M95I	-	-	-
141	SEQ	M	36-40	Urine	Nov-2020	S83I	G285A	M95I	-	-	-
142	SEQ	M	36-40	Urine	Nov-2020	S83I	G285A	M95I	-	-	-
143	SEQ	M	41-45	Urine	Jul-2020	D87N	WT	Wildtype	-	-	-
144	SEQ	M	26-30	Urine	Jul-2020	S83I	G285A	M95I	-	-	-
144.1	SEQ	M	26-30	Urine	May-2020	S83I	G285A	M95I	-	-	-
145	SEQ	M	21-25	Urine	Nov-2020	S83I	WT	Wildtype	-	-	-
146	SEQ	M	31-35	Urine	Jul-2019	Wildtype	C270T	Wildtype	-	-	-
147	SEQ	F	21-25	Cervical swab	Sep-2019	D87Y	WT	Wildtype	-	-	-
148	SEQ	M	41-45	Urine	Jul-2019	Wildtype	WT	Wildtype	-	-	-
149	SEQ	M	51-55	Urine	Jun-2019	D87N	WT	Wildtype	-	-	-
150	SEQ	M	26-30	Urine	Mar-2019	S83I	G285A	M95I	-	-	-
151	SEQ	M	>55	Urine	Apr-2019	S83I	G285A	M95I	-	-	-
152	SEQ	M	21-25	Urine	Dec-2018	Wildtype	WT	Wildtype	-	-	-
153	SEQ	M	26-30	Urine	Aug-2019	Wildtype	WT	Wildtype	-	-	-
154	SEQ	M	21-25	Urine	May-2018	Wildtype	WT	Wildtype	-	-	-
155	SEQ	M	31-35	Urine	Jan-2019	Wildtype	WT	Wildtype	-	-	-
156	SEQ	M	31-35	Urine	Feb-2019	Wildtype	WT	Wildtype	-	-	-
157	SEQ	F	26-30	Cervical swab	Aug-2019	Wildtype	WT	Wildtype	-	-	-
158	SEQ	F	21-25	Cervical swab	Nov-2018	Wildtype	WT	Wildtype	-	-	-
159	SEQ	M	21-25	Urine	Sep-2018	Wildtype	WT	Wildtype	-	-	-
160	SEQ	M	26-30	Urine	Sep-2018	Wildtype	WT	Wildtype	-	-	-

161	SEQ	M	36-40	Urine	Feb-2019	Wildtype	WT	Wildtype	-	-	-
161.1	SEQ	M	36-40	Penile swab	Feb-2019	Wildtype	WT	Wildtype	-	-	-
162	SEQ	M	36-40	Urine	Feb-2020	Wildtype	WT	Wildtype	-	-	-
163	SEQ	M	21-25	Urine	Jul-2018	S83I	G285A	M95I	-	-	-
163.1	SEQ	M	21-25	Urine	Aug-2018	S83I	G285A	M95I	-	-	-
164	SEQ	M	26-30	Urine	Mar-2019	Wildtype	WT	Wildtype	-	-	-
165	SEQ	F	26-30	Lower vaginal swab	Nov-2017	S83I	WT	Wildtype	-	-	-
166	SEQ	M	21-25	Urine	Aug-2018	Wildtype	WT	Wildtype	-	-	-
167	SEQ	F	18-20	Lower vaginal swab	Aug-2019	S83I	Undetermined	not characterised	-	-	-
168	SEQ	M	31-35	Urine	Jun-2020	D87N	WT	Wildtype	-	-	-
169	SEQ	M	26-30	Urine	Jan-2017	S83I	G285A	M95I	-	-	-
170	SEQ	M	36-40	Urine	Jul-2019	S83I	WT	Wildtype	-	-	-
171	SEQ	M	51-55	Urine	Dec-2018	Wildtype	WT	Wildtype	-	-	-
172	SEQ	F	26-30	Cervical swab	Nov-2020	S83I	WT	Wildtype	-	-	-
173	SEQ	M	21-25	Urine	Jun-2018	Wildtype	WT	Wildtype	-	-	-
174	SEQ	M	36-40	Urine	Feb-2019	S83I	WT	Wildtype	-	-	-
175	NQ	M	21-25	Urine	Jan-2019	Wildtype	WT	Wildtype	-	-	-
176	NQ	M	26-30	Urine	May-2019	D87N	WT	Wildtype	-	-	-
177	NQ	M	26-30	Urine	Jul-2019	S83R	WT	Wildtype	-	-	-
178	NQ	M	21-25	Urine	Jan-2021	S83I	WT	Wildtype	-	-	-
178.1	NQ	M	21-25	Urine	Dec-2020	Wildtype	WT	Wildtype	-	-	-
179	NQ	M	31-35	Urine	Jul-2019	S83I	Undetermined	not characterised	-	-	-
180	NQ	M	18-20	Urine	Jul-2020	S83I	G285A	M95I	-	-	-
181	NQ	M	21-25	Urine	May-2021	S83I	Undetermined	not characterised	-	-	-
182	NQ	F	21-25	High vaginal swab	Mar-2020	Wildtype	WT	Wildtype	-	-	-
183	NQ	M	21-25	Urine	Aug-2020	S83I	WT	Wildtype	-	-	-
184	NQ	F	31-35	Cervical swab	Sep-2020	Wildtype	WT	Wildtype	-	-	-
185	NQ	M	21-25	Urine	Jul-2020	Wildtype	WT	Wildtype	-	-	-
186	NQ	M	18-20	Urine	May-2019	S83I	G285A	M95I	-	-	-
187	NQ	M	36-40	Anal swab	Apr-2017	mixed WT / S83I	Undetermined	not characterised	-	-	-
188	NQ	F	21-25	Cervical swab	Jun-2019	S83I	WT	Wildtype	-	-	-
189	NQ	M	26-30	Urine	Oct-2020	D87N	WT	Wildtype	-	-	-
190	NQ	F	31-35	Cervical swab	Feb-2021	S83I	WT	Wildtype	-	-	-
191	NQ	M	41-45	Urethral swab	Jun-2019	Wildtype	WT	Wildtype	-	-	-
192	Other region not specified	F	26-30	unknown	Jul-2020	Wildtype	WT / G356A	Wildtype / G119D	-	-	-
193	Other region not specified	M	26-30	Urine	Apr-2020	S83I	WT	Wildtype	-	-	-
194	Other region not specified	M	26-30	Urine	Mar-2020	Wildtype	WT	Wildtype	-	-	-
195	Other region not specified	F	18-20	unknown	Jun-2019	Wildtype	WT	Wildtype	-	-	-
196	Other region not specified	F	26-30	Cervical swab	Mar-2017	G81C	WT	Wildtype	-	-	-
197	SEQ	M	46-50	Urine	Feb-2018	S83I	WT	Wildtype	-	-	-
198	SEQ	M	>55	Rectal swab	Oct-2018	D87Y	WT	Wildtype	-	-	-
199	SEQ	F	21-25	Urine	Sep-2017	S83I	WT	Wildtype	-	-	-
200	SEQ	F	21-25	Cervical swab	Apr-2017	D87N	WT	Wildtype	-	-	-
201	SEQ	F	31-35	Rectal swab	Apr-2017	S83I	WT	Wildtype	-	-	-
202	SEQ	M	31-35	Rectal swab	Jan-2017	mixed WT / S83I	WT	Wildtype	-	-	-
203	SEQ	M	36-40	Urine	Feb-2017	mixed WT / S83I	Undetermined	not characterised	-	-	-
204	SEQ	M	26-30	Urine	Oct-2017	S83I	WT	Wildtype	-	-	-
205	SEQ	M	36-40	Urine	Jan-2018	S83I	WT	Wildtype	-	-	-
205.1	SEQ	M	36-40	Urethral swab	Jan-2018	S83I	WT	Wildtype	-	-	-
205.2	SEQ	M	36-40	Urine	Feb-2018	S83I	WT	Wildtype	-	-	-
206	SEQ	M	36-40	Urine	Jun-2017	Wildtype	WT	Wildtype	-	-	-
207	NQ	F	21-25	Urine	Sep-2018	Wildtype	WT	Wildtype	-	-	-
208	NQ	M	21-25	Urine	Sep-2018	Wildtype	WT	Wildtype	-	-	-
209	SEQ	unknown	46-50	Urine	Aug-2019	S83I	WT	Wildtype	-	-	-
210	SEQ	M	26-30	Rectal swab	Feb-2019	Wildtype	WT	Wildtype	-	-	-
211	SEQ	F	31-35	Cervical swab	Dec-2020	S83I	G285A	M95I	-	-	-
212	SEQ	F	<18	Vaginal swab	Apr-2021	S83I	WT	Wildtype	-	-	-
213	SEQ	M	36-40	Urethral swab	Mar-2021	S83I	WT	Wildtype	-	-	-
214	SEQ	F	31-35	Vaginal swab	Oct-2020	S83I	G285A	M95I	-	-	-
215	SEQ	M	26-30	unknown	Feb-2021	S83I	WT	Wildtype	-	-	-
216	SEQ	M	26-30	Urethral swab	Jul-2020	S83I	WT	Wildtype	-	-	-
216.1	SEQ	M	26-30	Urine	Jul-2020	S83I	WT	Wildtype	-	-	-
217	SEQ	F	21-25	Vaginal swab	Mar-2021	Wildtype	WT	Wildtype	-	-	-
218	SEQ	F	26-30	Urine	Jan-2021	S83I	G285A	M95I	-	-	-
219	SEQ	F	36-40	Vaginal swab	Dec-2020	S83I	G285A	M95I	-	-	-
220	SEQ	M	31-35	Urine	Dec-2020	Wildtype	WT	Wildtype	-	-	-
221	SEQ	M	21-25	Urine	Nov-2020	Wildtype	WT	Wildtype	-	-	-
222	SEQ	M	26-30	Urine	Nov-2020	mutation not characterised	WT	Wildtype	78	8a	not characterised
223	SEQ	M	26-30	Rectal swab	Nov-2020	Wildtype	WT	Wildtype	-	-	-

224	SEQ	F	36-40	Urine	Apr-2021	S83I	Undetermined	not characterised	-	-	-
225	SEQ	F	21-25	High vaginal swab	Apr-2021	S83I	WT	Wildtype	-	-	-
226	SEQ	M	31-35	Urine	May-2017	S83I	WT	Wildtype	-	-	-
227	SEQ	M	41-45	Urine	Apr-2017	mixed WT / S83I	Undetermined	not characterised	-	-	-
228	NQ	M	21-25	Rectal swab	Feb-2019	S83I	WT	Wildtype	-	-	-
229	NQ	M	26-30	Urine	May-2018	S83I	G285A	M95I	-	-	-
230	NQ	M	21-25	Urine	Apr-2018	S83I	G285A	M95I	-	-	-
231	NQ	F	26-30	Urine	Aug-2017	S83I	WT	Wildtype	-	-	-
232	NQ	M	18-20	unknown	Nov-2017	mixed WT / S83I	WT	Wildtype	-	-	-
232.1	NQ	M	18-20	unknown	Oct-2017	S83I	WT	Wildtype	-	-	-
233	SEQ	F	31-35	Cervical swab	Jul-2018	D87Y	WT	Wildtype	-	-	-
234	SEQ	M	36-40	Urine	Mar-2017	mixed WT / S83I	WT	Wildtype	-	-	-
235	SEQ	M	>55	Rectal swab	Oct-2018	S83I	WT	Wildtype	-	-	-
236	SEQ	M	31-35	Urine	Jun-2019	Wildtype	Undetermined	not characterised	-	-	-
237	SEQ	M	>55	Rectal swab	Apr-2018	Wildtype	Undetermined	not characterised	-	-	-
238	SEQ	F	21-25	Cervical swab	Oct-2019	Wildtype	WT	Wildtype	-	-	-
239	SEQ	F	31-35	Vaginal swab	Sep-2020	S83I	G285A	M95I	-	-	-
240	SEQ	F	26-30	Vaginal swab	Oct-2020	S83I	G285A	M95I	-	-	-
241	SEQ	M	31-35	Urine	Aug-2020	S83I	WT	Wildtype	-	-	-
242	SEQ	M	21-25	Urine	Aug-2020	Wildtype	WT	Wildtype	-	-	-
243	SEQ	F	26-30	Vaginal swab	Jul-2019	S83I	G285A	M95I	-	-	-
244	SEQ	M	31-35	unknown	Aug-2019	S83I	WT	Wildtype	-	-	-
245	SEQ	M	51-55	Urine	Jun-2020	Wildtype	WT	Wildtype	-	-	-
246	SEQ	M	31-35	Urine	Jun-2020	S83I	G285A	M95I	-	-	-
247	SEQ	M	26-30	Urine	Apr-2020	Wildtype	WT	Wildtype	-	-	-
248	SEQ	F	21-25	Vaginal swab	Jun-2020	S83I	WT	Wildtype	-	-	-
248.1	SEQ	F	21-25	Cervical swab	Oct-2020	S83I	WT	Wildtype	-	-	-
249	SEQ	F	31-35	Urine	Mar-2020	S83I	G285A	M95I	-	-	-
250	SEQ	M	41-45	Urine	Feb-2020	S83I	WT	Wildtype	-	-	-
251	SEQ	F	18-20	Cervical swab	Jan-2019	Wildtype	WT	Wildtype	-	-	-
252	SEQ	M	>55	Rectal swab	Apr-2019	Wildtype	WT / C272T	Wildtype / P91L	-	-	-
253	SEQ	M	31-35	Urine	Oct-2019	Wildtype	WT	Wildtype	-	-	-
254	SEQ	F	31-35	Urine	Jun-2019	Wildtype	WT	Wildtype	-	-	-
255	SEQ	F	18-20	Vaginal swab	May-2019	Wildtype	WT	Wildtype	-	-	-
256	SEQ	F	18-20	No site specified	May-2019	S83I	WT	Wildtype	-	-	-
256.1	SEQ	F	18-20	Vaginal swab	Aug-2019	S83I	WT	Wildtype	-	-	-
256.2	SEQ	F	18-20	Vaginal swab	Aug-2019	S83I	WT	Wildtype	-	-	-
257	SEQ	M	26-30	Urine	May-2019	S83I	G285A	M95I	-	-	-
257.1	SEQ	M	26-30	Urine	Jul-2019	S83I	G285A	M95I	-	-	-
258	SEQ	M	26-30	Urine	Sep-2019	D87N	WT	Wildtype	-	-	-
259	SEQ	M	26-30	Urine	Jun-2019	S83I	G285A	M95I	-	-	-
259.1	SEQ	M	26-30	Urethral swab	Oct-2019	S83I	G285A	M95I	-	-	-
259.2	SEQ	M	26-30	Urine	Oct-2019	S83I	G285A	M95I	-	-	-
260	SEQ	F	31-35	Vaginal swab	Apr-2019	Wildtype	WT	Wildtype	-	-	-
261	SEQ	M	21-25	Rectal swab	Feb-2020	S83I	WT	Wildtype	-	-	-
262	SEQ	M	36-40	Urine	Apr-2018	S83I	WT	Wildtype	-	-	-
263	SEQ	M	21-25	Rectal swab	Apr-2019	D87N	WT	Wildtype	-	-	-
264	SEQ	M	26-30	Rectal swab	Oct-2019	D87N	WT	Wildtype	-	-	-
265	SEQ	M	36-40	Urine	Sep-2019	Wildtype	WT	Wildtype	-	-	-
266	SEQ	M	>55	Rectal swab	Feb-2020	S83I	WT	Wildtype	-	-	-
267	SEQ	M	21-25	Penile swab	Jan-2020	D87N	WT	Wildtype	-	-	-
267.1	SEQ	M	21-25	Urine	May-2020	D87N	WT	Wildtype	-	-	-
268	SEQ	M	21-25	Urine	Oct-2019	Wildtype	WT	Wildtype	-	-	-
269	SEQ	M	51-55	Urine	Sep-2018	S83I	Undetermined	not characterised	-	-	-
270	SEQ	M	21-25	Urine	Apr-2019	Wildtype	WT	Wildtype	-	-	-
271	SEQ	M	31-35	Urine	Jan-2020	S83I	G285A	M95I	-	-	-
271.1	SEQ	M	31-35	Urine	Jun-2019	S83I	WT	Wildtype	-	-	-
272	SEQ	M	21-25	Urine	Apr-2020	S83I	WT	Wildtype	-	-	-
273	SEQ	M	36-40	Urine	Apr-2018	mixed WT / S83I	WT	Wildtype	-	-	-
274	SEQ	M	26-30	Rectal swab	Feb-2020	Wildtype	WT	Wildtype	-	-	-
275	SEQ	M	31-35	Urine	Jul-2019	Wildtype	WT	Wildtype	-	-	-
276	SEQ	M	41-45	Urine	Sep-2019	Wildtype	WT	Wildtype	-	-	-
277	SEQ	M	26-30	Urine	Jan-2019	S83I	WT	Wildtype	-	-	-
278	SEQ	M	51-55	Rectal swab	Sep-2019	S83I	WT	Wildtype	-	-	-
279	SEQ	M	36-40	Rectal swab	May-2019	Wildtype	WT	Wildtype	-	-	-
280	NQ	F	<18	Lower vaginal swab	Jan-2017	Wildtype	WT	Wildtype	-	-	-
281	NQ	F	21-25	Urine	Jul-2019	S83I	G285A	M95I	-	-	-
282	NQ	F	21-25	Vaginal swab	Sep-2019	S83I	G285A	M95I	-	-	-
283	NQ	F	21-25	Vaginal swab	Oct-2019	Wildtype	WT	Wildtype	-	-	-

284	NQ	M	18-20	Urine	Jul-2020	S83I	G285A	M95I	-	-	-
285	NQ	F	21-25	Vaginal swab	Jul-2020	S83I	G285A	M95I	-	-	-
286	NQ	M	26-30	Urine	Oct-2020	S83I	G285A	M95I	-	-	-
286.1	NQ	M	26-30	Urine	Jul-2020	S83I	G285A	M95I	-	-	-
287	NQ	F	18-20	Cervical swab	Jan-2021	S83I	WT	Wildtype	-	-	-
288	NQ	M	26-30	Urine	Apr-2020	Wildtype	WT	Wildtype	-	-	-
289	NQ	M	26-30	Urine	Jan-2020	S83I	G285A	M95I	-	-	-
290	NQ	M	21-25	Urine	Apr-2020	Wildtype	WT	Wildtype	-	-	-
291	NQ	M	21-25	Urine	Jan-2020	Wildtype	WT	Wildtype	-	-	-
292	NQ	F	18-20	Urine	Jun-2019	S83I	G285A	M95I	-	-	-
293	NQ	F	18-20	Cervical swab	Apr-2019	S83I	G285A	M95I	-	-	-
294	NQ	M	18-20	Urine	Mar-2019	S83I	G285A	M95I	-	-	-
295	NQ	M	21-25	Urine	Nov-2018	S83I	G285A	M95I	-	-	-
296	NQ	M	21-25	Urine	Jun-2019	S83I	G285A	M95I	-	-	-
297	NQ	F	<18	High vaginal swab	Jul-2019	S83I	G285A	M95I	-	-	-
297.1	NQ	F	<18	Vaginal swab	Oct-2019	S83I	G285A	M95I	-	-	-
298	NQ	F	21-25	Cervical swab	Jan-2021	S83I	G285A	M95I	-	-	-
299	NQ	M	18-20	Urine	May-2020	Wildtype	WT	Wildtype	-	-	-
300	NQ	F	26-30	Anal swab	Oct-2019	S83I	G285A	M95I	-	-	-
301	NQ	M	21-25	Urine	Sep-2019	Wildtype	WT	Wildtype	-	-	-
302	NQ	M	18-20	Urine	Oct-2019	S83I	G285A	M95I	-	-	-
303	NQ	M	36-40	Anal swab	Jul-2017	S83I	WT	Wildtype	-	-	-
304	NQ	M	41-45	Anal swab	Jul-2017	S83I	WT	Wildtype	-	-	-
305	NQ	M	21-25	Urine	Jul-2019	S83I	G285A	M95I	-	-	-
306	NQ	M	26-30	Urine	Mar-2020	S83I	G285A	M95I	-	-	-
307	NQ	M	21-25	Urine	Jan-2020	S83I	G285A	M95I	3	Undetermined	not characterised
307.1	NQ	M	21-25	Urine	Oct-2019	mutation not characterised	Undetermined	not characterised	-	-	-
308	NQ	F	21-25	Cervical swab	May-2019	S83I	G285A	M95I	-	-	-
309	NQ	F	26-30	Cervical swab	Feb-2020	S83I	G285A	M95I	-	-	-
310	NQ	M	36-40	Anal swab	Mar-2017	D87N	WT	Wildtype	-	-	-
310.1	NQ	M	36-40	Anal swab	Feb-2017	D87N	WT	Wildtype	-	-	-
311	NQ	F	21-25	Vaginal swab	Sep-2020	S83R	WT	Wildtype	-	-	-
312	NQ	F	<18	Vaginal swab	May-2017	S83I	WT	Wildtype	-	-	-
313	NQ	M	31-35	Anal swab	Apr-2017	S83I	WT	Wildtype	-	-	-
314	NQ	M	21-25	Urine	Apr-2019	S83I	G285A	M95I	-	-	-
314.1	NQ	M	21-25	Urine	Jul-2019	mutation not characterised	Undetermined	not characterised	-	-	-
315	NQ	M	21-25	Urine	Jun-2019	S83I	G285A	M95I	-	-	-
316	NQ	M	21-25	unknown	Jul-2019	mutation not characterised	Undetermined	not characterised	-	-	-
317	NQ	M	26-30	Urine	Mar-2021	S83I	WT	Wildtype	-	-	-
318	NQ	F	31-35	Anal swab	Jan-2021	S83I	WT	Wildtype	-	-	-
319	NQ	M	46-50	Anal swab	May-2017	Wildtype	WT	Wildtype	-	-	-
320	NQ	M	41-45	Urine	Oct-2020	S83I	G285A	M95I	-	-	-
321	NQ	F	41-45	Vaginal swab	Oct-2018	D87N	WT	Wildtype	-	-	-
322	NQ	F	18-20	Genital swab	Apr-2020	Wildtype	WT	Wildtype	-	-	-
323	NQ	F	21-25	Urine	Feb-2019	S83I	Undetermined	not characterised	-	-	-
324	unknown	unknown	unknown	unknown	unknown	S83I	G285A	M95I	-	-	-
325	unknown	unknown	unknown	Urine	unknown	S83I	WT	Wildtype	-	-	-
326	NQ	F	<18	Urine	Jul-2019	Wildtype	WT	Wildtype	-	-	-

SEQ = South East Queensland, NQ = North Queensland, M = male, F = female, WT = wildtype
AA ParC mutation = Amino acid change of the ParC mutation determined by PCR and Sanger sequencing
DNA / AA GyrA mutation = DNA / Amino acid change of the GyrA mutation determined by PCR and Sanger Sequencing
Sample ID numbering is based on individual patients (e.g. 1, 2, 3) with additional samples collected from the same patient over time expressed as respective decimal numbers (e.g. 1.1, 1.2, 1.3)
* "mixed" susceptibility populations are highlighted in light blue with the mutations found in those samples seperated by a /
